# Identification of three conserved linear B cell epitopes on the SARS-CoV-2 spike protein

**DOI:** 10.1080/22221751.2022.2109515

**Published:** 2022-09-14

**Authors:** Aiping Wang, Yuanyuan Tian, Hongliang Liu, Peiyang Ding, Yumei Chen, Chao Liang, Yongkun Du, Dawei Jiang, Xifang Zhu, Jiajia Yin, Gaiping Zhang

**Affiliations:** a School of Life Sciences, Zhengzhou University, Zhengzhou, People’s Republic of China; b Longhu Laboratory of Advanced Immunology, Zhengzhou, People’s Republic of China; c Henan Key Laboratory of Immunobiology, Zhengzhou, People’s Republic of China; d College of Veterinary Medicine, Henan Agricultural University, Zhengzhou, People’s Republic of China; e School of Advanced Agricultural Sciences, Peking University, Beijing, People’s Republic of China

**Keywords:** SARS-CoV-2, spike protein, B cell epitope, monoclonal antibodies, conserved epitope

## Abstract

Spike (S) glycoprotein is the most significant structural protein of SARS-CoV-2 and a key target for neutralizing antibodies. In light of the on-going SARS-CoV-2 pandemic, identification and screening of epitopes of spike glycoproteins will provide vital progress in the development of sensitive and specific diagnostic tools. In the present study, NTD, RBD, and S2 genes were inserted into the pcDNA3.1(+) vector and designed with N-terminal 6× His-tag for fusion expression in HEK293F cells by transient transfection. Six monoclonal antibodies (4G, 9E, 4B, 7D, 8F, and 3D) were prepared using the expressed proteins by cell fusion technique. The characterization of mAbs was performed by indirect -ELISA, western blot, and IFA. We designed 49 overlapping synthesized peptides that cover the extracellular region of S protein in which 6 amino acid residues were offset between adjacent (S1–S49). Peptides S12, S19, and S49 were identified as the immunodominant epitope regions by the mAbs. These regions were further truncated and the peptides S12.2 ^286^TDAVDCALDPLS^297^, S19.2 ^464^FERDISTEIYQA^475^, and S49.4 ^1202^ELGKYEQYIKWP^1213^ were identified as B- cell linear epitopes for the first time. Alanine scans showed that the ^D^467, ^I^468, ^E^471, ^Q^474, and ^A^475 of the epitope S19.2 and ^K^1205, ^Q^1208, and ^Y^1209 of the epitope S49.4 were the core sites involved in the mAbs binding. The multiple sequence alignment analysis showed that these three epitopes were highly conserved among the variants of concern (VOCs) and variants of interest (VOIs). Taken together, the findings provide a potential material for rapid diagnosis methods of COVID-19.

## Introduction

Coronavirus disease 2019 (COVID-19) pandemic has damaged the lives many of people severely and presented a major threat to public hygiene across the globe [1,2]. By the end of 11 March 2020, the World Health Organization had declared COVID-19 to be a public health emergency of a global concern pandemic [3]. As of 5 March 2022, more than 557 million cases had been confirmed with over 6.3 million deaths across the world [4]. It is a respiratory infectious disease characterized as severe respiratory distress syndrome caused by SARS-CoV-2 [5].

SARS-CoV-2 is a kind of enveloped coronavirus distributed in the cytoplasm. The genome length of SARS-CoV-2 was 29.9 kb and mainly codes four structural proteins, including spike (S) glycoprotein, small envelope (E) glycoprotein, membrane (M) glycoprotein, and nucleocapsid (N) protein, as well as several auxiliary proteins [6,7]. The S protein is a transmembrane protein with a molecular weight of approximately 150 kDa and forms a homologous trimer on the virus surface readily, which promotes the binding of the virus to host cells through the attraction of angiotensin-converting enzyme 2 (ACE2) [8,9]. N protein is a highly phosphorylated structural protein. It binds RNA and is involved in the process associated with viral replication [10–12]. The M protein is related to determining the shape of the virus envelope. The E protein is the smallest and plays a role in the emergence and process of the maturation of the virus [13].

S protein is a highly glycosylated protein, which is composed of 1273 amino acids with 21–35 N-terminal glycosylation sites. It has receptor binding and membrane fusion activities and is closely related to virus infection and pathogenesis. With the action of protease, S protein was cleaved into two segments S1 and S2. Among them, S1 is highly variable, with significant variation among different strains of the virus, including the surface viral receptor-binding domain (RBD) that binds to host cells. COVID-19 can spread from species to person and from person to person because it has the RBD that can perfectly bind ACE2. While S2 is relatively conserved and mainly participates in the process of virus and cell membrane fusion, S1 can be divided into two domains: the N-terminal domain (NTD) and the C-terminal domain (CTD). Most NTD binds to sugar receptors and CTD binds to protein receptors. The receptor ACE2 and the receptor DPP4 of MERS-CoV both bind to CTD. This domain is also an important neutralizing region and is a key target for vaccine development [13].

Epitopes are the basis of protein antigenicity, the special chemical groups in antigen molecules that determine antigen specificity, and the basic structure and function units of antigen molecules that induce a specific immune response. Therefore, accurate and detailed mapping of antigen epitopes is of positive significance to the diagnosis of diseases, the targeted modification of protein molecules to reduce the immunogenicity of protein drugs, the design of artificial vaccines with no side effects, and immune intervention therapy [14,15]. B-cell epitopes are clusters of amino acids on the surface of antigen molecules that can be specifically recognized and bound to secreted antibodies and B-cell receptors to induce cellular and humoral immune responses in the host. Identification of B-cell epitope has many important biological significances, such as deepening the understanding of immune response and autoimmune diseases, providing candidate epitopes for the development of the establishment of disease diagnosis methods and helping to reveal the mechanism of action of therapeutic antibodies [16,17].

In this study, we successfully generated six monoclonal antibodies using proteins expressed in the eukaryotic system. Three novel conserved B-cell epitopes were identified by overlapping peptides scanning using mAbs. The epitope information identified in this study may be of great significance for serological detection.

## Materials and methods

### Gene, cell, and serum

The S protein gene sequence was obtained from NCBI (GenBank accession number MN908947.3). According to the S protein gene sequence, we synthesized the S protein plasmid by Sangon Biotech (Shanghai) Co., Ltd. The human embryonic kidney 293T (HEK293T), HEK293F cells, and hybridoma cells sp2/0 were preserved in our laboratory. The HEK293T cell was cultured in DMEM medium (Gibco, Cat. No. A4192101, USA) supplement with 8% fetal bovine serum (Transgen, Cat. No. FS301-02, China) and 293F cells were cultured in SMM 293-TII Expression Medium (Sino Biological, Cat. No. M293TII). The sp2/0 cell was maintained in RPMI 1640 medium (Gibco, Cat. No. 11875093, USA) supplement with 8% fetal bovine serum (Transgen, Cat. No. FS301-02, China). The *Escherichia coli* DH5α competent cells were purchased from TransGen Biotech (Transgen, Cat. No. CD201-01, China).

### Preparation of target protein

By analyzing the structure of S protein, we divided the protein into three segments for expression, NTD, RBD, and S2. The three target proteins were obtained by PCR from the pUC57-S plasmid (synthesized by Sangon, Shanghai) using three pairs of primers (pCNTD-F/pCNTD-R, pCRBD-F/pCRBD-R, pCS2-F/pCS2-R) including *BamHI/XbaI* restriction enzyme cutting sites (Table S1). The recombinant plasmids, pcDNA3.1-NTD, pcDNA3.1-RBD, and pcDNA3.1-S2 were designed with an N-terminal 6× His label and constructed by three PCR amplification products inserted in the pcDNA3.1 (+) vector by ligase and then the connecting products were transformed into *E. coli*. DH5α competent cells. The recombinants were amplified and extracted using the EndoFree Plasmid Maxi Kit (CWBIO, Cat. No. CW2104, China).

The protein was produced by HEK293F cells. Specifically, the HEK293F cells were cultured in SMM 293-TII Expression Medium (Sino Biological, Cat. No. M293TII, China) and the cells in a logarithmic growth phase with viability higher than 90%. Then cell density was adjusted to 3.0–5.0 × 10^6^, and the system of transfection solution followed the instructions of SMM 293-TII with the transfection reagent Sinofection (Sino Biological, Cat. No. SdTF02, China). The HEK293F cells were collected for protein identification 48–72 h after transfection. The cell precipitates were harvested by centrifugation and lytic cell protein via sonication with protease inhibitors. Then proteins were purified by Ni Sepharose™ affinity chromatography column according to the references [18].

### Animal immunization strategies and preparation of monoclonal antibodies

Nine female BALB/C mice aged six to eight weeks were randomly divided into three groups of three. The first group was immunized with NTD protein, the second with RBD protein, and the third with S2 protein. Specifically, the protein antigen was emulsified with Freund’s Adjuvant Complete to an oil-in-water state. Each mouse was immunized with a subcutaneous multipoint injection of 200 μl of an emulsified mixture of antigens including 10 μg protein. After a three-week interval, booster immunization was replaced with an emulsified mixture of the immune antigen protein and Freund’s Adjuvant Incomplete for each mouse at the same dose of 200 μl, including 10 μg protein. The mice were immunized booster twice, since the second immunization, the tail blood was gathered every other week for the measurement of titer. Mice with the highest serum titer were selected for intraperitoneal booster immunization and prepared for cell fusion.

Three days after intraperitoneal immunization, blood samples were collected from eyeballs as positive serum. Spleen was extracted from mice and prepared into single spleen cells on 200 mesh screened. The well-growing hybridoma cells sp2/0 about 2–5 × 10^7^ were collected. The spleen cells and SP2/0 cells were washed by GNK (0.8% NaCl, 0.04% KCl, 0.2% glucose, 0.001% phenol red, 0.356% Na_2_HPO_4_·12H_2_O, 0.078% NaH_2_PO_4_·2H_2_O) medium and mixed together. After the cell clusters dispersed, 50% PEG1500 1 ml was added slowly, and the reaction was terminated. The cells were suspended in the selective medium RPMI 1640 8% FBS containing HAT and placed in 96-well cell culture plates, which were incubated in an incubator containing 5% CO_2_ at 37°C. About seven days after the fusion culture, cell clusters could be seen and the production of cell antibodies was detected by indirect ELISA. The positive wells were transferred to 48-well cell plates and half of the medium was replaced with RPMI 1640 8% FBS containing HT. The culture was continued and tested again until overgrown. Repeated detection three times, positive antibody cell lines were selected for limited dilution subcloning for monoclonal antibody screening. The monoclonal cell lines were enlarged and cultivated and the mAbs were prepared by the induction of ascites in vivo.

### Screening and characterization of monoclonal antibodies

Indirect enzyme-linked immunosorbent assay (i-ELISA) was used for screening the positive antibody cell lines and testing the titer of monoclonal antibodies. The purified NTD, RBD, and S2 proteins were coated in the ELISA plates, respectively at 4°C overnight. The plates were blocked with 5% skim milk in PBST (PBS with 0.05% Tween-20) at 37°C for 2 h [19]. The anti-NTD, RBD, S2 protein antibodies cell supernatants, and dilution ascites were performed as primary antibodies. The Goat Anti-Mouse IgG/HRP (Solarbio, Cat. No. SE131, China) was used as the secondary antibody, and washed three times by PBST. Adding the TMB chromogenic solution to estimate the ability of antibodies with proteins, 2 M H_2_SO_4_ was used as the stop reaction fluid. Then the OD value at 450 nm was performed by a multi-functional enzyme standard instrument (Omega, Germany).

Western blotting was performed on the reactivity of monoclonal antibodies with NTD, RBD, and S2 proteins and the expression of these proteins was identified. SDS-PAGE was performed on target proteins and the protein gels were transferred to the PVDF membrane which was blocked at 37°C for 1 h, the anti-NTD, RBD, and S2 protein mAbs were incubated as the primary antibodies at 37°C for 1 h. After being washed six times by PBST, the Goat Anti-Mouse IgG/HRP (Solarbio, Cat. No. SE131, China) was used as the secondary antibody at 37°C for 1 h. The membrane was washed six times and the Enhanced Chemiluminescent (ECL) reagent (NCM Biotech, Cat. No. P2300, China) was used to detect the binding ability between the target proteins and anti-target protein mAbs.

An immunofluorescence assay (IFA) experiment was used to verify the specificity of the monoclonal antibodies with NTD, RBD, and S2 proteins. The HEK293T cells were cultured in a 12-well plate in a DMEM medium (Gibco, Cat No. A4192101, USA) supplement with 8% FBS. The constructed recombinant plasmid pcDNA3.1-NTD, pcDNA3.1-RBD, and pcDNA3.1-S2 were transfected into 293T cells by Lipofectamine™ 2000 (Invitrogen, Cat. No. 11668027, USA) in which the cell density reached 70–80%. The 293T cell supernatant was discarded after transfection 24–48h and the cells were fixed with 4% paraformaldehyde for 10 min. The cell plate was washed by PBS three times and blocked at 37°C for 1 h and incubated with anti-NTD, anti-RBD, and anti-S2 mAbs as the primary antibodies at room temperature for 1 h. Then the plate was washed three times with PBS and incubated with Alexa Fluor 488 Donkey anti-Mouse IgG (H+L) (Invitrogen, Cat. No. A-21202, USA) at room temperature for 1 h. Then the cells were coloured by DAPI (Solarbio, Cat. No. C0060, China) and viewed under an electron fluorescence microscope [20].

### Design and synthesis of polypeptides

In this study, 49 overlapping peptides with 6 amino acid offsets were designed to cover the extracellular region of S protein (GenBank access number: MN908947.3) (Table S2). In order to map the peptides screened in this study more accurately, the peptides were further truncated. Cysteine (C) was added to the N-terminus of each truncated peptide sequence to conjugate BSA protein. All the peptides designed in this study were synthesized by GL Biochem (China). The purity of peptides was all greater than 95%.

### Identification and screening of polypeptides

Peptide-ELISA was performed to map the peptide. Briefly, the peptides or peptides coupled with BSA by the coupling agent Sulfo-SMCC (Thermo Scientific, Cat. No. 22322, USA) coated in the 96-well ELISA plates (WUXI GUOSHENG BIO-ENGINEERING, China) in carbonate buffer at a concentration of 4 µg/ml and 100 µl/well at 4°C overnight [19]. Then the plates were blocked at 37°C for 2 h. The anti-NTD, RBD, and S2 mAbs were added at 1:5000 dilution in blocking buffer as the primary antibodies. Then the secondary antibody the Goat Anti-Mouse IgG/HRP (Solarbio, Cat. No. SE131, China) was used to detect the peptide-bound mAbs at room temperature for 1 h. Finally, 100 µl/well TMB substrate was added to develop and 50 µl/well 2 M H_2_SO_4_ was used as the stop buffer. The OD value at 450 nm was performed using the microplate reader [21] (Omega, Germany).

Dot-blot was conducted to determine the fine peptide of the SARS-CoV-2 S protein. The peptides coupled with BSA were pointed onto the Nitrocellulose membrane (NC) and blocked at 37°C for 1 h. After that, the membranes were incubated with the anti-NTD, anti-RBD, and anti-S2 mAbs prepared in this study at 1:10,000 dilution in blocking buffer as the primary antibodies. The Goat Anti-Mouse IgG/HRP (Solarbio, Cat No. SE131, China) was used as the secondary antibody at room temperature for 1 h. The Enhanced Chemiluminescent (ECL) reagent (NCM Biotech, Cat. No. P2300, China) was used to detect the binding ability between the target proteins and anti-target protein mAbs.

### Alanine-scanning mutagenesis

Alanine mutation was used to determine the crucial sites of amino acids of peptides S19.2 and S49.4. The amino acid sequence of the peptides was substituted with alanine (A) one by one. At the same time, the alanine (A) in the sequence was substituted with glycine (G). The coupled peptides were coated on the 96-well ELISA plates, peptide S19.2 and peptide S49.4 were used as a positive control in their mutant groups. The monoclonal antibodies 4B, 7D, and 8F, 3D were used as primary antibodies, and the Goat Anti-Mouse IgG/HRP (Solarbio, Cat No. SE131, China) was used as the secondary antibody.

### Conservatism and spatial structure analysis of epitopes

The conservatism of the peptide sequences screened by the mAbs in this study was carried out by the multiple sequence alignment of S protein strains from the GISAID database (https://www.gisaid.org/) using the Jalview software (Version 2.11.1.4) [22]. The tertiary structure of proteins was gathered from the PDB database. The PyMOL software (Version 2.5.2, Schrödinger, LLC) was used to visualize the spatial distribution of epitopes identified by mAbs prepared in this research [23].

## Results

### Protein construction, expression, and purification

**Figure 1.**
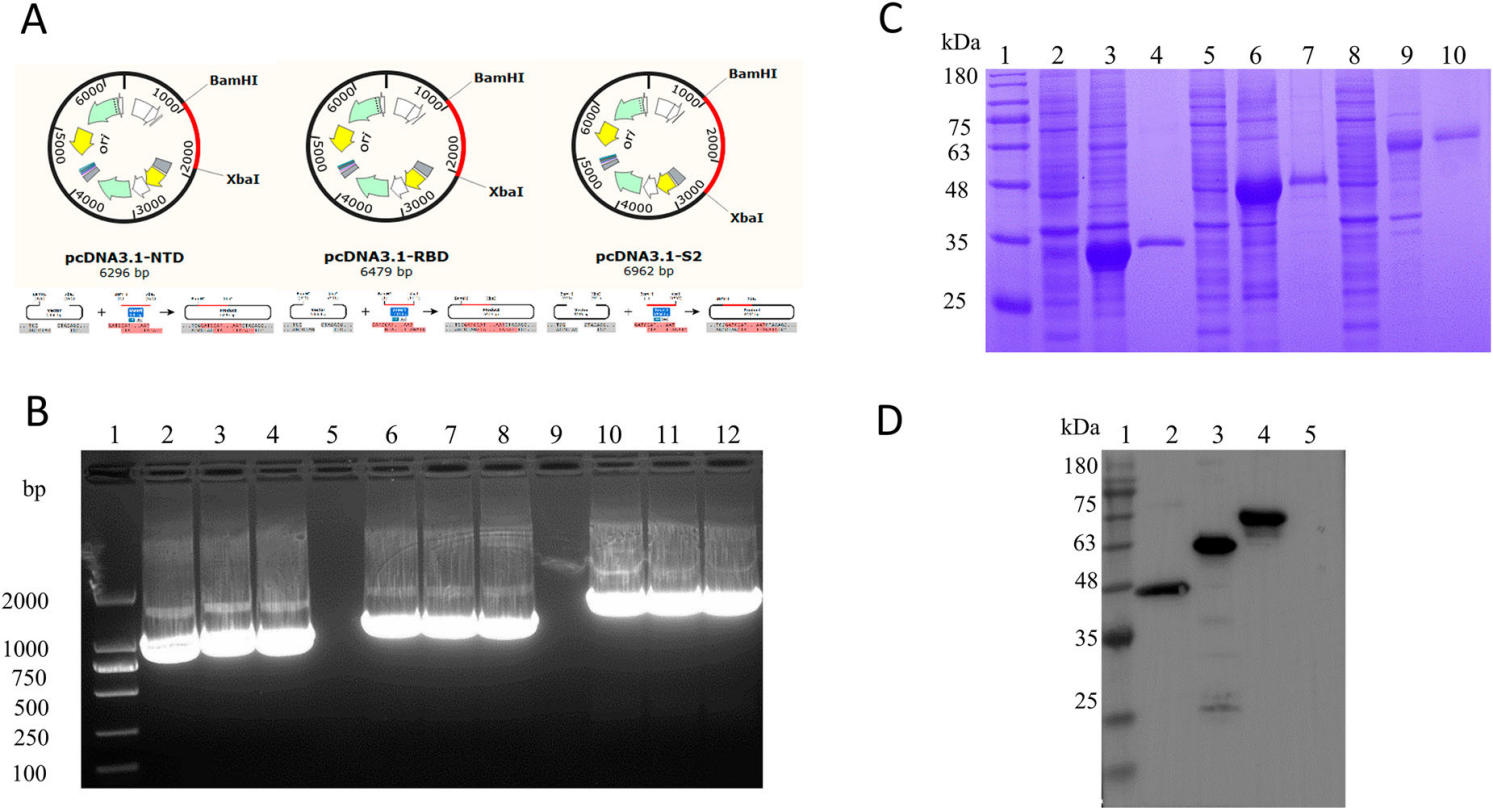
Preparation of the target protein. (A) Strategies for the construction of NTD, RBD, and S2 protein. (B) Identification of target fragments. Lane 1, DNA Marker. Lane 2–4, Amplicons of the NTD gene. Lane 5 and 9, Blank lane. Lane 6–8, Amplicons of the RBD gene. Lane 10–12, Amplicons of the S2 gene. (C) SDS-PAGE for the identification of the expression and purification of the proteins NTD, RBD, and S2. Lane 1, Protein Marker. Lane 2, NTD cell supernatant. Lane 3, NTD cell precipitate. Lane 4, NTD-purified. Lane 5, RBD cell supernatant. Lane 6, RBD cell precipitate. Lane 7, RBD-purified. Lane 8, S2 cell supernatant. Lane 9, S2 cell precipitate. Lane 10, S2-purified. (D) Western Blot for the identification of the proteins NTD, RBD, and S2 with HRP-Conjugated 6*His-Tag mAbs. Lane 1, Protein Marker. Lane 2, NTD purified. Lane 3, RBD purified. Lane 4, S2 purified. Lane 5, the blank vector pcDNA3.1.

Based on the extracellular region nucleotide sequence of S protein, we designed and synthesized (by Sangon, Shanghai) three pairs of primers and constructed three segments: NTD, RBD, and S2. The construction of the recombinant plasmids was in accordance with the principles of plasmid restructuring (Figure 1(A)). The PCR bands of the NTD, RBD, and S2 were about at 920 bp, 1100 bp, and 1500 bp (Figure 1(B)), respectively, and the results were in accordance with the theoretical length. At the same time, the fragments of insertion were identified by sequencing and were consistent with the theoretical sequence. The recombinant positive plasmids were transfected into HEK293F cells following the instructions. The HEK293F cells were collected 72 h after transfection. The proteins were collected in the cell precipitate. After purification by the Ni affinity chromatography column, the obtained proteins were identified by SDS-PAGE and the results showed that the recombinant proteins (NTD, RBD, and S2) had distinct bands at 35, 40, and 58 kDa (Figure 1(C)). The results of Western Blot were also evident that there are obvious bands at 35, 46, and 60 kDa and consistent with the theoretical size of the proteins, but not for the negative empty vector pcDNA3.1 (+) (Figure 1(D)).

### Preparation of monoclonal antibodies

BLAB/C mice were immunized according to the immunization procedure (Figure S1(A)). A tail blood sample was collected to determine the serum titers against NTD, RBD, and S2 proteins of the mice two weeks after the third immunization. The mouse NTD-2, mouse RBD-3, and mouse S2-3 showed a higher titer than another two mice (Figure S1(B)). Therefore, these three mice in each group were selected for further mAbs preparation. Hybridoma cell lines with the secretion of stable targeting NTD, RBD, and S2 were screened by indirect-ELISA. Six mAbs hybridoGma cell lines were obtained by limited dilution subcloning and named 4G (anti-NTD), 9E (anti-NTD), 4B (anti-RBD), 7D (anti-RBD), 8F (anti-S2), 3D (anti-S2).

### Identification and characterization of monoclonal antibodies

**Figure 2.**
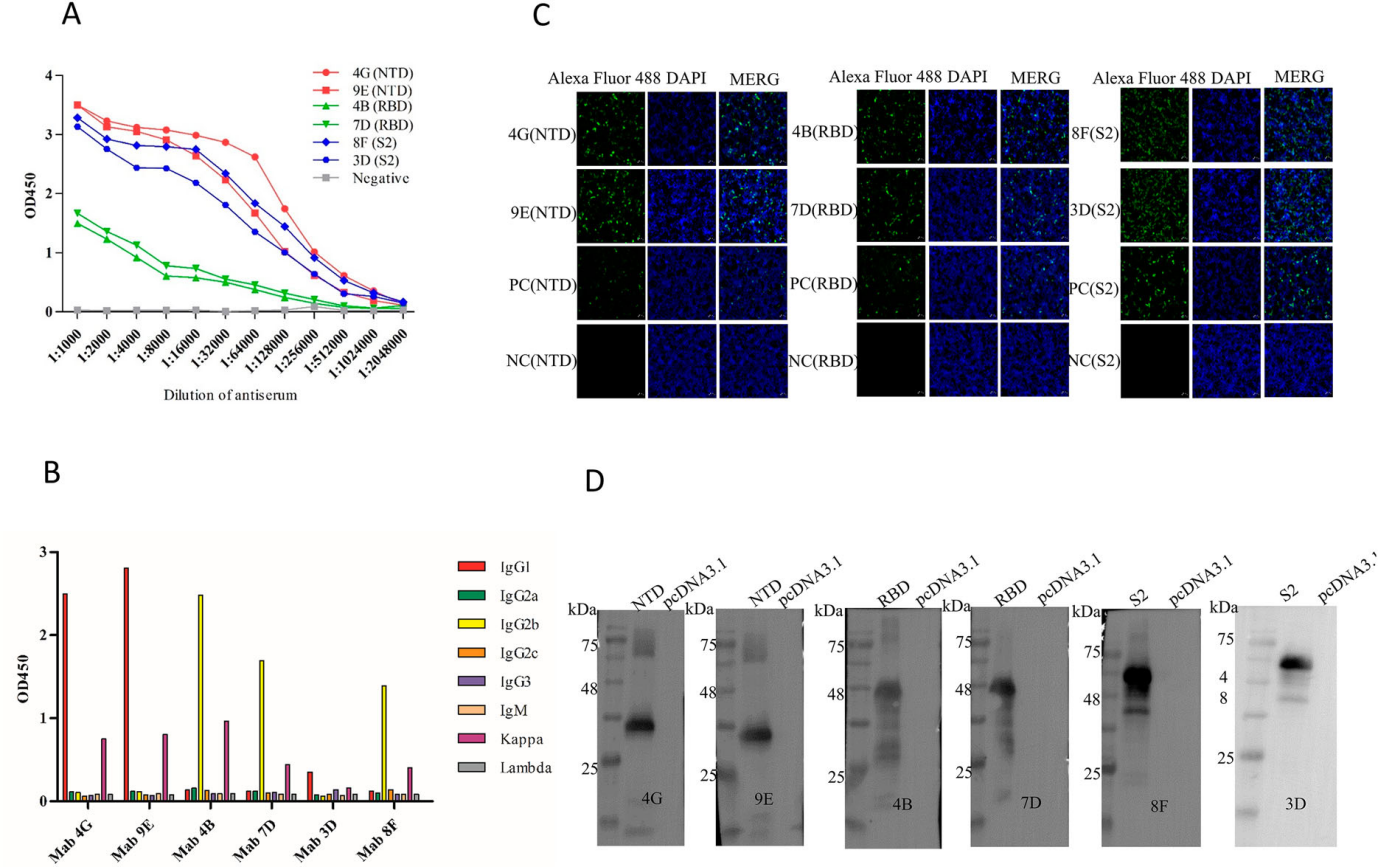
Identification of mAbs prepared in this study. (A) Ascites titers of monoclonal antibodies performed by the indirect ELISA. (B) The subtype of monoclonal antibodies. (C) The IFA assay was used to analyze the reactivity between mAbs screened and NTD, RBD, and S2 proteins. The green colour is the reaction of anti-NTD, RBD, and S2 mAbs with corresponding proteins. The blue colour is the nucleus of the HEK293T cells. The negative control is the untransfected cells incubated with the eyeball blood (positive serum). The positive control is transfected pcDNA3.1-NTD, pcDNA3.1-RBD, and pcDNA3.1-S2 cells that are incubated with the eyeball blood. (D) Western blot was used to perform the reactivity between proteins (NTD, RBD, and S2) and mAbs (prepared in this study).

A large number of monoclonal antibodies were produced by ascite induction. The ascitic fluid titers of anti-NTD and anti-S2 mAbs can reach 1:1,024,000, and anti-RBD mAbs can reach 1:25,600 in this study by indirect-ELISA (Figure 2(A) and Table 1). The i-ELISA was used to analyze the affinity constant of these six mAbs, the high-affinity parameter “Ka” of some mAbs can reach about 5.81 × 10^9^ calculated as in the following equation (Table 1) [24,25]. The IFA experimental results also proved that the mAbs produced can specifically recognize NTD, RBD, and S2 proteins (Figure 2(C)). Among them, S2 protein reacted best with corresponding monoclonal antibodies (8F and 3C). The results of Western blot showed that the monoclonal antibodies (4G, 9E, 4B, 7D, 8F, and 3D) all can recognize the corresponding target proteins (NTD, RBD, and S2), implied all the monoclonal antibodies prepared in this study identified the linear epitopes (Figure 2(D)). In addition, the mAbs subtypes were determined mainly including the IgG1, IgG2b, and Kappa by the commercial kit (Figure 2(B)).

$$a=\frac{n-1}{2(n[Ab^{\prime }]t-[Ab]t)}$$

$$n=\frac{[Ag]t}{[{Ag}^{\prime }]t}$$

[Ab′] and [Ab] are the mAb concentrations (ng/L) that correspond to 50% of maximum absorption values of two concentration plate-coating antigens.

**Table 1.** Characteristics of mAbs.

MAbs	Immune protein	Epitope	MAbs subtypes	Titres		Affinity constant “Ka”(L/mol)
				Supernatants	Ascitic fluid	
4G	NTD	Linear	IgG1, Kappa	1:6400	1:1,024,000	4.70 × 107
9E	NTD	Linear	IgG1, Kappa	1:6400	1:1,024,000	9.61 × 108
4B	RBD	Linear	IgG2b, Kappa	1:800	1:256000	1.06 × 109
3D	RBD	Linear	IgG2b, Kappa	1:800	1:256,000	5.81 × 109
8F	S2	Linear	IgG2b, Kappa	1:12800	1:1,024,000	1.68 × 108
7D	S2	Linear	IgG1, Kappa	1:6400	1:1,024,000	7.14 × 107

[Ag]*t* and [Ag′]*t* represent the molarity of two t coating sources.

### Identify the binding region of antigen and mAbs

**Figure 3.**
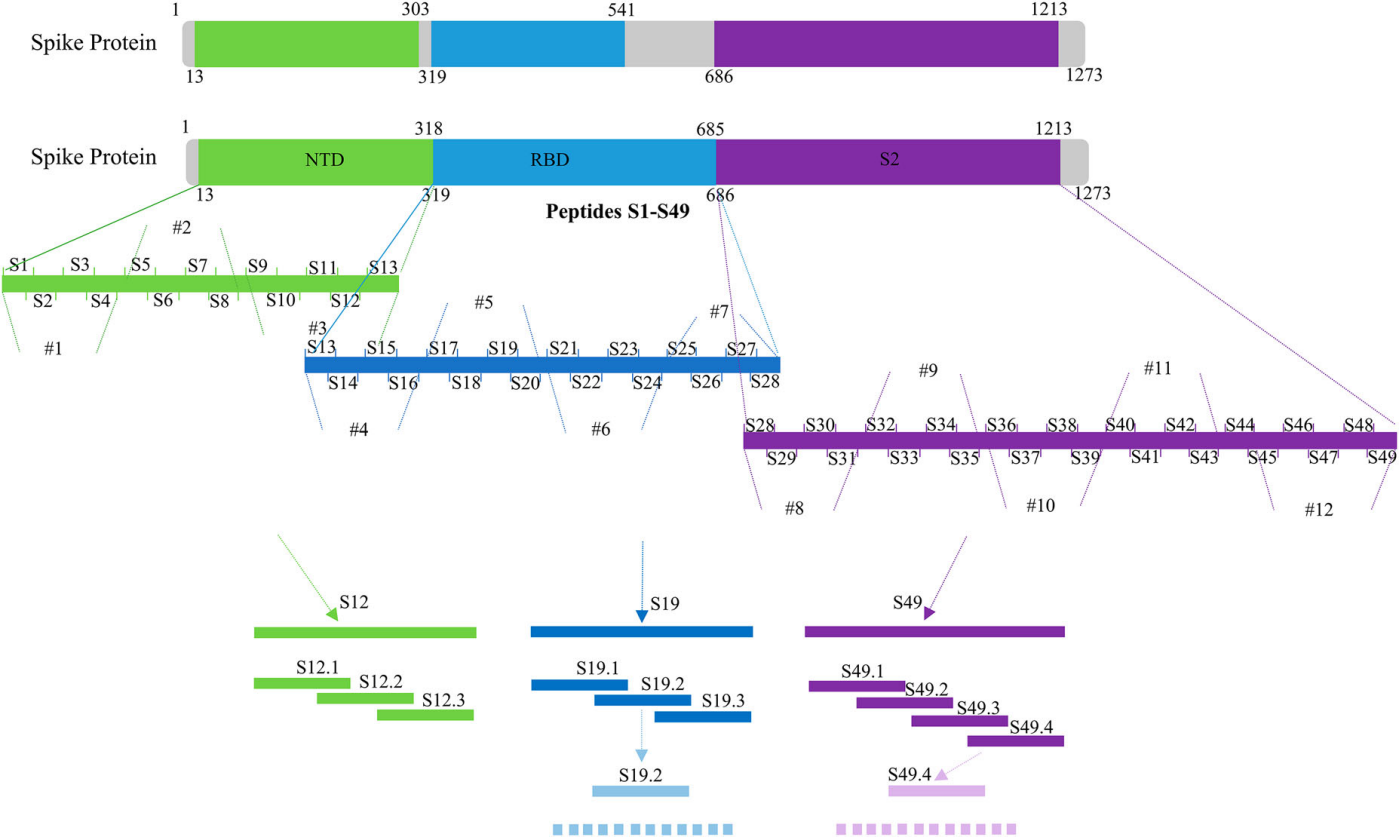
Framework for the design of epitope screened (#1–#12 were the peptide pool designs).

**Figure 4.**
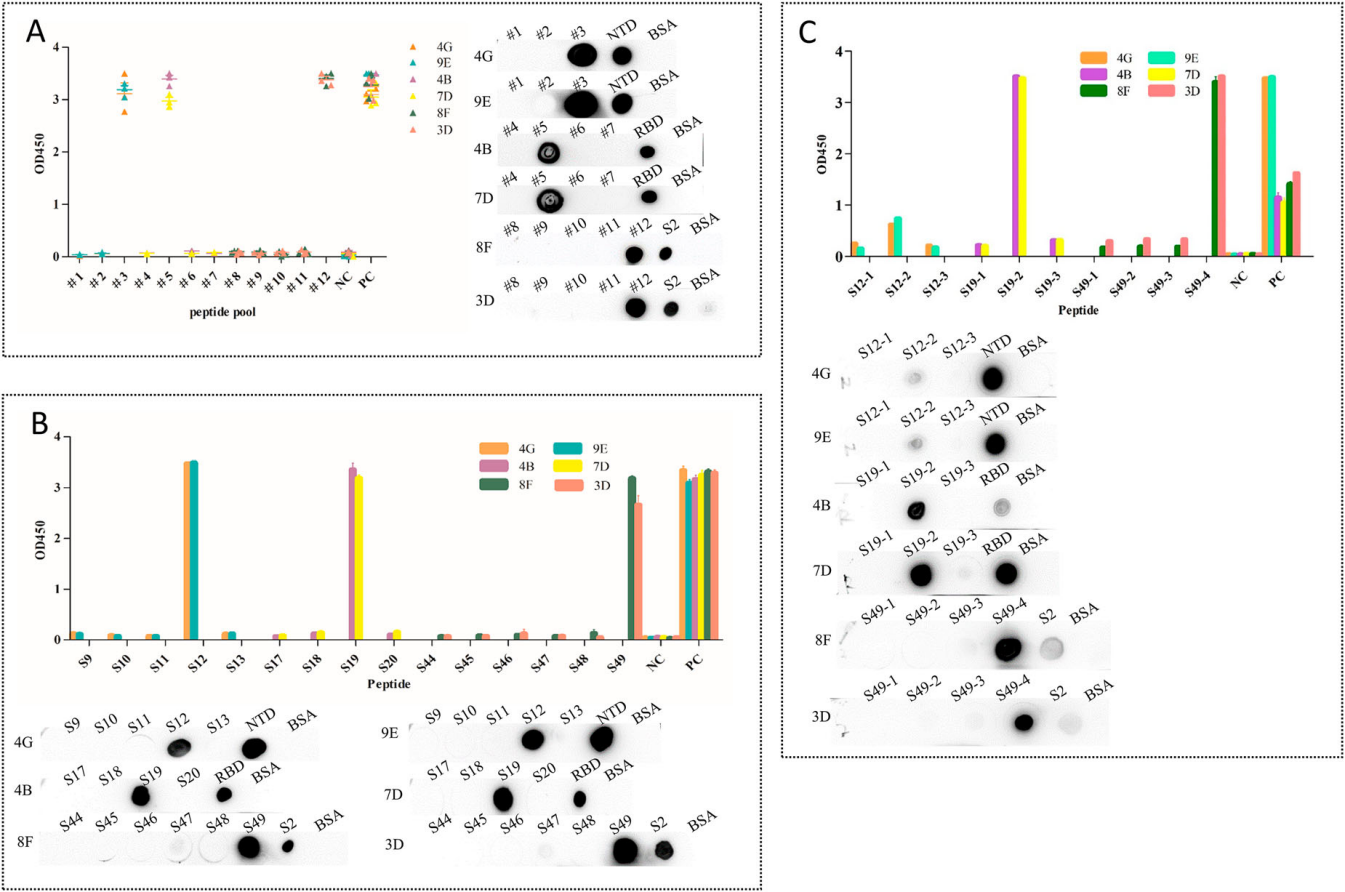
Map of the linear epitope. Negative Control (NC): BSA. (A) The peptide pools spanning the extracellular region of the S protein were screened by the indirect ELISA and dot immunoblotting. (Distribution of peptide pools was as in Table S3). (B) The peptides S9–S13, S17–S20, and S44–S49 were performed to react with the mAbs (4G, 9E, 4B, 7D, 8F, and 3D) by the indirect ELISA and dot immunoblotting. (C) Map of the truncated sequence of peptides S12, S19, and S49 by the indirect ELISA and dot immunoblotting.

In order to determine the main area of antigen and mAbs binding, we designed 49 overlapping synthesized peptides with 6 amino acid offsets covering the extracellular domain sequence of the protein (Figure 3 and Table S3). Peptides were divided into 12 peptide pools (Table S3), wherein #1, #2, and #3 spanning NTD protein and were detected by mAbs 4G and 9E; #4, #5, #6, and #7 spanning RBD protein and were detected by mAbs 4B and 7D; #8, #9, #10, #11, and #12 spanning S2 protein and were detected by mAbs 8F and 3D (Figure 4). The results of peptide -ELISA and dot-bolt all showed that peptide pool #3 was recognized by mAbs 4G and 9E, #5 was recognized by mAbs 4B and 7G, and #12 was recognized by mAbs 8F and 3D (Figure 4(A)). Further experimental verification showed that S12, S19, and S49 were specific peptides recognized by mAbs (Figure 4(B)). The peptides were further truncated to determine the key regions recognized by mAbs (Table S4). The experimental results indicated that the amino acid residues 286–297 (S12.2) could be recognized by mAbs 4G and 9E, 464–475 (S19.2) recognized by mAbs 4B and 7D, 1202–1213 (S49.4) recognized by mAbs 8F and 3D (Figure 4(C)).

### Identification of the critical amino acid residues of the epitopes

**Figure 5.**
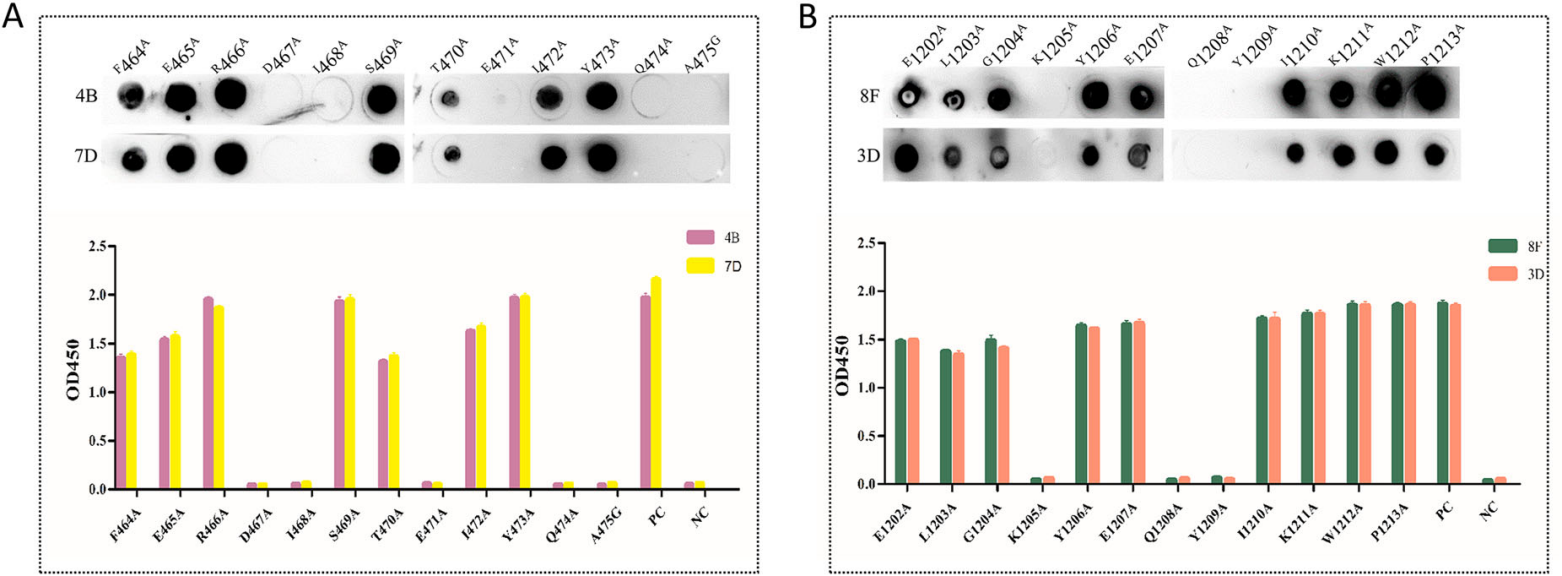
Alanine scanning mutation was used to identify the key amino acid sites in mAbs binding. (A) The binding ability of mutation sequences of peptide S19.2 to mAbs 4B and 7D. (B) The binding ability of mutation sequences of peptide S49.4 to mAbs 8F and 3D.

Alanine scanning was used to further identify the critical sites of the epitopes identified in this study. According to the above results, the peptide S12.2 reacted weakly with the mAbs 4G and 9E, while the peptides S19.2 and S49.4 reacted strongly with the mAbs 4B, 7D, 8F, and 3D. Therefore, we performed the identification of the key amino acids of peptides S19.2 and S49.4. Based on the amino acid sequence of the peptides, a panel of alanine/glycine-substituted sequences for the peptides S19.2 and S49.4 was designed (Table 2). The results showed that the ^467^D, ^468^I, ^471^E, ^474^Q, and ^475^A had no reaction with the responding mAbs when these residues were substituted with the alanine/glycine, while it could be for the other positions of an amino acid (Figure 5(A)). It could be inferred that residues ^467^D, ^468^I, ^471^E, ^474^Q, and ^475^A were critical at the binding among the peptide S19.2 and mAbs 4B and 7D. Similarly, the ^1205^K, ^1208^Q, and ^1209^Y were the critical residues in the peptide S49.4 (Figure 5(B)).

**Table 2.** Alanine-scanning mutagenesis of peptides S19.2 and S49.4.

S19.2	Sequence	S49.4	Sequence
F464A	AERDISTEIYQA	E1202A	ALGKYEQYIKWP
E465A	FARDISTEIYQA	L1203A	EAGKYEQYIKWP
R466A	FEADISTEIYQA	G1204A	ELAKYEQYIKWP
D467A	FERAISTEIYQA	K1205A	ELGAYEQYIKWP
I468A	FERDASTEIYQA	Y1206A	ELGKAEQYIKWP
S469A	FERDIATEIYQA	E1207A	ELGKYAQYIKWP
T470A	FERDISAEIYQA	Q1208A	ELGKYEAYIKWP
E471A	FERDISTAIYQA	Y1209A	ELGKYEQAIKWP
I472A	FERDISTEAYQA	I1210A	ELGKYEQYAKWP
Y473A	FERDISTEIAQA	K1211A	ELGKYEQYIAWP
Q474A	FERDISTEIYAA	W1212A	ELGKYEQYIKAP
A475G	FERDISTEIYQG	P1213A	ELGKYEQYIKWA

### Conservative analysis of epitopes

**Figure 6.**
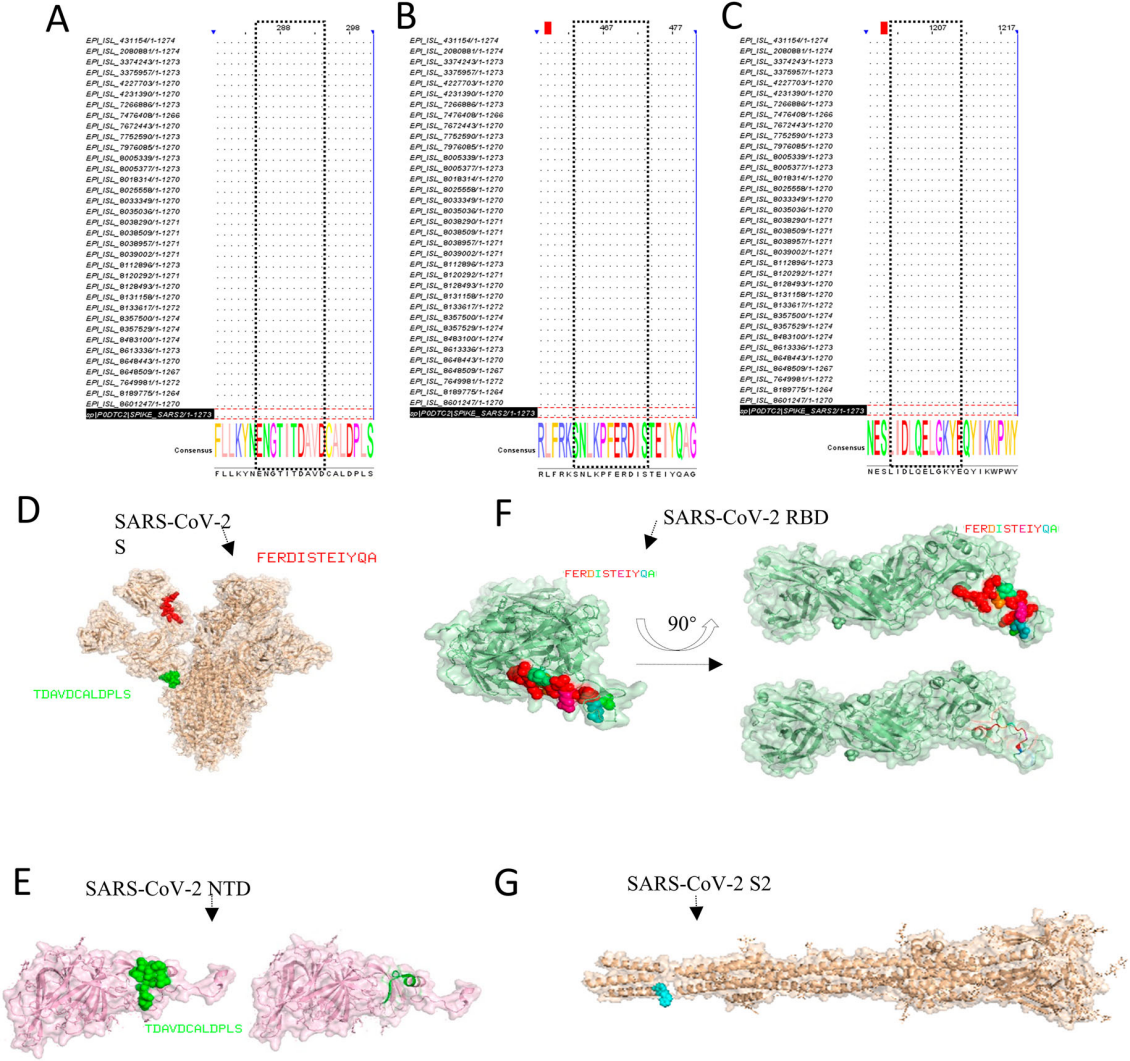
Bioinformatics analysis of epitopes. The epitopes were performed to multiple sequence alignments with the sequence of partial VOC and VOI strains. (A) S12.2 286TDAVDCALDPLS297, (B) S19.2 464FERDISTEIYQA475, (C) S49.4 1202ELGKYEQYIKWP1213). The spatial structure of epitopes was screened in this research. (D) The epitope 286TDAVDCALDPLS297 (Green colour) and epitope 464FERDISTEIYQA475 (red colour) are shown on the S protein (PBD: 7CWU). (E) The characteristics of epitope 286TDAVDCALDPLS297 (Green colour) on the spatial structure of the NTD protein (PBD: 7B62). (F) The characteristics of the epitope 464FERDISTEIYQA475 on the spatial structure of the RBD protein (PBD: 6W41). (G) The partial region (blue colour) of the epitope 1202ELGKYEQYIKWP1213 is shown on the spatial structure of the S2 protein (PBD: 7E9T).

The conservatism of antigen – mAbs-binding regions (epitopes) was also analyzed. We mainly compared the strain sequences of variants of concern and variants of interest of different countries of the S proteins. The information about the strains of S protein in this study is in Table S5, and the GenBank accession number of expressed proteins in this study is MN908947.3. The results of sequence alignment are shown that the epitope ^286^TDAVDCALDPLS^297^ was very conservative among all strains about the VOCs and VOIs (Figure 6(A)). The epitope ^464^FERDISTEIYQA^475^ and ^1202^CELGKYEQYIKWP^1213^ also were conservative (Figure 6(B,C)). Among them, it contains the latest mutation of the strain Omicron. Consequently, the monoclonal antibodies prepared in this study are highly conservative, which provides a feasible strategy and choice for the establishment of detection methods.

### Spatial structure and position analysis of epitopes

The visualization of identification of epitopes’ spatial structure in this research was performed by the software PyMOL. The epitopes S12.2 ^286^TDAVDCALDPLS^297^, and S19.2 ^464^FERDISTEIYQA^475^ were at the surface of the S protein (PBD: 7CWU) (Figure 6(D)). Among them, the epitope ^286^TDAVDCALDPLS^297^ was exposed as the α-helix on the secondary structure of NTD protein (PBD: 7B62) (Figure 6(E)) and the epitope ^464^FERDISTEIYQA^475^ was approximately located at the loop of the RBD protein (PBD: 6W41) (Figure 6(F)). As the epitope ^1202^CELGKYEQYIKWP^1213^ is at the end of the S2 protein sequence, the complete spatial structure of the location of the epitope at protein cannot be displayed. Only part of the amino acid sequence can be displayed in spatial structure, the amino acids were also located at the surface of the S2 protein (PBD: 7E9T) (Figure 6(G)). The above results of the spatial structure further demonstrated that the identified epitopes are linear and immunodominant.

## Discussion

The novel pandemic has been posing a huge threat to people’s lives and health [1]. Among the SARS-CoV-2 structural proteins mediates virus invasion into target cells and induces immune response [7]. It is also the largest structural protein of the virus. It plays an important role in the infection of the target cells by the virus. Meanwhile, it is crucial that the S protein induces immunity generating neutralizing antibody responses [26]. It is the main protein in the study of major targets for serological diagnosis. Screening out the S protein immunodominant epitope region is of great value for the diagnosis of the disease. In this research, the eukaryotic expression system HEK293F was used to produce the NTD, RBD, and S2 proteins, six specific monoclonal antibodies were screened and generated by the cell fusion technique. Three linear epitopes were identified by monoclonal antibodies and the key amino acid sites and conservation of epitopes was also discussed.

Monoclonal antibodies that specifically recognize the S protein play a key role in basic research and are important materials for diagnostic tools. The key to the preparation of specific monoclonal antibodies is the antigen with high biological activity and high purity. In this study, the HEK293F cells were used to produce the antigen NTD, RBD, and S2 proteins. The HEK293F cells were a fast and intuitive transfection system with all post-translational modifications and cofactors for protein function. HEK293F cells, as the expression system, produced high bioactivity proteins as antigens and prepared six monoclonal antibodies with specific binding antigens. Wherein, the titers of mAbs 4G, 9E, 8F, and 7D can reach 1:102400 (Figure 2(A)). At the same time, mAbs prepared in this research can especially recognize the target protein antigens via the IFA assay and Western blotting (Figure 2(C,D)). It indicated that antigens with well spatial conformation and biological activity are crucial for the preparation of monoclonal antibodies.

Epitopes, also known as antigen epitopes, are a part of an antigen molecule that stimulates the body to produce antibodies and can be recognized with antibodies and they are generally composed of 5–15 amino acid residues and are the material basis for stimulating specific immune responses [27]. Epitopes include linear and conformational ones. The identification and analysis of antigenic epitopes are important for understanding the structure and function of antigenic substances. It is of great significance to calculate safe and develop epitope-based diagnostic reagents. To identify the B-cell immune-dominant epitopes, a panel of continuous overlapping synthesized peptides covering the extracellular region of the S protein were designed, and the ability of binding with the mAbs prepared in this study was tested by dot-blot and peptide ELISA. The results showed that the peptides S12, S19, and S49 showed a strong reaction with mAbs (4G, 9E), (4B, 7D), (8F and 3D). Next, the synthesized peptides of 12 amino acids were designed. The experimental results of this study showed that the binding sites of S12.2 with mAbs were not obvious, indicating that S12.2, recognized by antibodies 4G and 9E, was not the dominant epitope, but it could react strongly with S12 of 30 amino acids. Epitopes S19.2 and S49.4 can react strongly with corresponding monoclonal antibodies 4B, 7D, 8F, and 3D, indicating that they are immune-dominant epitopes.

In order to map the key amino acid of peptides S19.2 and S49.4 in antibody binding, a series of peptide amino acid-specific mutation sequences were designed (Table 2). Amino acids in peptide sequences were mutated to alanine (A) one by one, while alanine (A) was mutated to glycine (G). From the results, the binding ability of the peptide to mAbs 4B and 7D disappeared after the mutation of amino acid residues positions of ^D^467, ^I^468, ^E^471, ^Q^474, and ^A^475. It revealed that these positions were key amino acid residues binding to antibodies. In the same way, the positions of ^K^1205, ^Q^1208, and ^Y^1209 were the key amino acid residues in binding with mAbs 8F and 3D.

We further analyzed the conservatism and spatial distribution of the epitopes. We compared the epitope sequence of strains from VOCs and VOIs of different countries and found that S12.2, S19.2, and S49.4 were very conserved among strains. The PyMOL software was also used to show the distribution of the spatial structure of the epitope, S12.2 was found as the α-helix on the secondary structure of the NTD protein, and the epitope ^464^FERDISTEIYQA^475^ was approximately located at the loop of the RBD protein and part of the S49.2 could be displayed on the surface. These results further suggest that these epitopes are dominant B-cell linear epitopes.

B-cell epitopes of the SARS-CoV-2 S protein have also been reported previously. Among them, Polyiam et al. employed the immunoinformatic tool BepiPred-2.0 server predicted the liner B-cell epitope 455–478 within the RBD domain [28]; Dawood et al. predicted B-cell epitopes 273–293, 468–488 using the IEDB software [29]; Kesarwani et al. reported the B-cell epitope 863–1205, 1207–1246 using the Bepipred 2.0 server [30]; Ras-Carmona et al. predicted the epitope 456–475 using the BCEPS server [31]. These articles predicted B-cell linear epitopes using bioinformatics methods, some of the predicted epitopes overlapped with those identified in this study. This further confirms that the B-cell epitopes identified in this study are immunodominant. In addition, Jiang et al. reported that the epitope 473–479 was identified by monoclonal antibodies produced by immunizing mice with peptides [23]; Epitopes 455–469 can elicit a robust immune response in mice reported by Lu et al. [32]. Lan et al. mapped the neutralizing antibody epitope including the ^T^470 [33]. These reports verified B-cell epitopes in the RBD region via experiments. Among them, a little overlapping with the epitope was identified in this study. Other reports on B-cell epitopes such as Poh et al. reported two neutralized linear B-cell epitopes identified by sera from COVID-19 patients covering the positions 562–580, 820–835 [26]; Zhang et al. identified the epitope 1031–1045 by convalescent serum [34]; Lu et al. identified conserved epitope 864–882 in convalescent COVID-19 patients [35]. B-cell epitopes reported in these articles are different from those in this study, which proves that the epitopes in this study are novel B-cell epitopes and can be predicted by bioinformatics methods, further indicating that these epitopes are immunodominant.

In summary, the proteins NTD, RBD, and S2 were constructed, expressed, and purified. Via HEK293F cells expressed proteins as antigens, six monoclonal antibodies were prepared by the cell fusion technique. Three novel B-cell epitopes, S12.2, S19.2, and S49.4, were screened by monoclonal antibodies, which were particularly conserved among the VOC and VOI strains. The results of alanine-scanning mutagenesis showed that the ^D^467, ^I^468, ^E^471, ^Q^474, and ^A^475 of the peptide S19.2 and the ^K^1205, ^Q^1208, ^Y^1209 of the peptides of S49.2 were the key sites involved in the mAbs binding. The findings of this study provide some basis for further analysis of the structure and function of the S protein.

## Supplementary Material

Supplemental Material

## Data Availability

The data that support for the findings of this study are all contained in the manuscript and supplementary materials.
