# Supplementary material for: Identification of three conserved linear B cell epitopes on the SARS-CoV-2 spike protein
Source: Emerg Microbes Infect. 2022 Sep 14;11(1):2120–31. doi: 10.1080/22221751.2022.2109515 (PMC9487943; doi:10.1080/22221751.2022.2109515)
Supplement: Supplemental Material [file TEMI_A_2109515_SM3034.docx]

**Table S1** Primers are designed to construct proteins.

| Name for primers | Sequence for primers (5’-3’) |
| --- | --- |
| pCNTD-F | CGC*GGATCC*ATGCACCACCACCACCATCACTCTCAGTGCGTTAACCTCACC |
| pCNTD-R | GC*TCTAGA*TTAGAAGTTGCTGGTCTGGTAGAT |
| pCRBD-F | CGC*GGATCC*ATGCACCACCACCACCATCACCGTGTCCAACCTACTGAGTCT |
| pCRBD-R | GC*TCTAGA*TTACCTGGCTCTACGGGGACTGT |
| pCS2-F | CGC*GGATCC*ATGCACCACCACCACCATCACTCTGTTGCTAGCCAGTCAATC |
| pCS2-F | GC*TCTAGA*TTATGGCCACTTAATGTACTGCTCGT |

**Table S2** Design of overlapped peptides cover the extracellular domain of S protein in this research.

| Name | Sequence | Name | Sequence |
| --- | --- | --- | --- |
| S1 | SQCVNLTTRTQLPPAYTNSFTRGVYYPDKV | S26 | AIHADQLTPTWRVYSTGSNVFQTRAGCLIG |
| S2 | YYPDKVFRSSVLHSTQDLFLPFFSNVTWFH | S27 | AGCLIGAEHVNNSYECDIPIGAGICASYQT |
| S3 | NVTWFHAIHVSGTNGTKRFDNPVLPFNDGV | S28 | CASYQTQTNSPRRARSVASQSIIAYTMSLG |
| S4 | PFNDGVYFASTEKSNIIRGWIFGTTLDSKT | S29 | YTMSLGAENSVAYSNNSIAIPTNFTISVTT |
| S5 | TLDSKTQSLLIVNNATNVVIKVCEFQFCND | S30 | TISVTTEILPVSMTKTSVDCTMYICGDSTE |
| S6 | FQFCNDPFLGVYYHKNNKSWMESEFRVYSS | S31 | CGDSTECSNLLLQYGSFCTQLNRALTGIAV |
| S7 | FRVYSSANNCTFEYVSQPFLMDLEGKQGNF | S32 | LTGIAVEQDKNTQEVFAQVKQIYKTPPIKD |
| S8 | GKQGNFKNLREFVFKNIDGYFKIYSKHTPI | S33 | TPPIKDFGGFNFSQILPDPSKPSKRSFIED |
| S9 | SKHTPINLVRDLPQGFSALEPLVDLPIGIN | S34 | RSFIEDLLFNKVTLADAGFIKQYGDCLGDI |
| S10 | LPIGINITRFQTLLALHRSYLTPGDSSSGW | S35 | DCLGDIAARDLICAQKFNGLTVLPPLLTDE |
| S11 | DSSSGWTAGAAAYYVGYLQPRTFLLKYNEN | S36 | PLLTDEMIAQYTSALLAGTITSGWTFGAGA |
| S12 | LKYNENGTITDAVDCALDPLSETKCTLKSF | S37 | TFGAGAALQIPFAMQMAYRFNGIGVTQNVL |
| S13 | CTLKSFTVEKGIYQTSNFRVQPTESIVRFPNITNLCPFGE | S38 | VTQNVLYENQKLIANQFNSAIGKIQDSLSS |
| S14 | LCPFGEVFNATRFASVYAWNRKRISNCVAD | S39 | QDSLSSTASALGKLQDVVNQNAQALNTLVK |
| S15 | SNCVADYSVLYNSASFSTFKCYGVSPTKLN | S40 | LNTLVKQLSSNFGAISSVLNDILSRLDKVE |
| S16 | SPTKLNDLCFTNVYADSFVIRGDEVRQIAP | S41 | RLDKVEAEVQIDRLITGRLQSLQTYVTQQL |
| S17 | VRQIAPGQTGKIADYNYKLPDDFTGCVIAW | S42 | YVTQQLIRAAEIRASANLAATKMSECVLGQ |
| S18 | GCVIAWNSNNLDSKVGGNYNYLYRLFRKSN | S43 | ECVLGQSKRVDFCGKGYHLMSFPQSAPHV |
| S19 | LFRKSNLKPFERDISTEIYQAGSTPCNGVE | S44 | SAPHGVVFLHVTYVPAQEKNFTTAPAICHD |
| S20 | PCNGVEGFNCYFPLQSYGFQPTNGVGYQPY | S45 | PAICHDGKAHFPREGVFVSNGTHWFVTQRN |
| S21 | VGYQPYRVVVLSFELLHAPATVCGPKKSTN | S46 | FVTQRNFYEPQIITTDNTFVSGNCDVVIGI |
| S22 | PKKSTNLVKNKCVNFNFNGLTGTGVLTESN | S47 | DVVIGIVNNTVYDPLQPELDSFKEELDKYF |
| S23 | VLTESNKKFLPFQQFGRDIADTTDAVRDPQ | S48 | ELDKYFKNHTSPDVDLGDISGINASVVNIQ |
| S24 | AVRDPQTLEILDITPCSFGGVSVITPGTNT | S49 | SVVNIQKEIDRLNEVAKNLNESLIDLQELGKYEQYIKWP |
| S25 | TPGTNTSNQVAVLYQDVNCTEVPVAIHADQ |  |  |

**Table S3** Design of Peptide pools

| Peptide pools No. | Peptide sequence distribution |
| --- | --- |
| #1 | S1, S2, S3, S4 |
| #2 | S5, S6, S7, S8 |
| #3 | S9, S10, S11, S12, S13 |
| #4 | S13, S14, S15, S16 |
| #5 | S17, S18, S19, S20 |
| #6 | S21, S22, S23, S24 |
| #7 | S25, S26, S27, S28 |
| #8 | S28, S29, S30, S31 |
| #9 | S32, S33, S34, S35 |
| #10 | S36, S37, S38, S39 |
| #11 | S40, S41, S42, S43, S44 |
| #12 | S45, S46, S47, S48, S49 |

**Table S4** Amino acid truncated sequence of of peptide S12, S19 and S49

| Name | Sequence | Name | Sequence | Name | Sequence |
| --- | --- | --- | --- | --- | --- |
| S12.1 | LKYNENGTITDA | S19.1 | LFRKSNLKPFER | S49.1 | SVVNIQKEIDRL |
| S12.2 | TDAVDCALDPLS | S19.2 | FERDISTEIYQA | S49.2 | DRLNEVAKNLNE |
| S12.3 | PLSETKCTLKSF | S19.3 | YQAGSTPCNGVE | S49.3 | LNESLIDLQELG |
|  |  |  |  | S49.4 | ELGKYEQYIKWP |

**Table S5** The sequences information of conservation of epitope analysis was used in this study.

The accession number of sequences was obtained from GISAID database.

| Sequence Accession Number | Pango lineage | WHO Label | Country |
| --- | --- | --- | --- |
| EPI_ISL_8035036 | B.1.1.7 | Alpha | USA |
| EPI_ISL_8018314 | B.1.1.7 | Alpha | Switzerland |
| EPI_ISL_8025558 | B.1.1.7 | Alpha | Zambia |
| EPI_ISL_4231390 | B.1.1.7 | Alpha | Norway |
| EPI_ISL_4227703 | B.1.1.7 | Alpha | Germany |
| EPI_ISL_8033349 | B.1.351 | Beta | France |
| EPI_ISL_7976085 | B.1.351 | Beta | USA |
| EPI_ISL_7672443 | B.1.351 | Beta | Italy |
| EPI_ISL_7266886 | B.1.351 | Beta | India |
| EPI_ISL_7649981 | B.1.351 | Beta | Namibia |
| EPI_ISL_8005377 | P.1 | Gamma | Brazil |
| EPI_ISL_8005339 | P.1 | Gamma | Brazil |
| EPI_ISL_8112896 | P.1 | Gamma | Netherlands |
| EPI_ISL_3375957 | P.1 | Gamma | Peru |
| EPI_ISL_3374243 | P.1 | Gamma | USA |
| EPI_ISL_8120292 | B.1.617.2 | Delta | USA |
| EPI_ISL_8039002 | B.1.617.2 | Delta | Germany |
| EPI_ISL_8038957 | B.1.617.2 | Delta | Switzerland |
| EPI_ISL_8038509 | B.1.617.2 | Delta | Italy |
| EPI_ISL_8038290 | B.1.617.2 | Delta | France |
| EPI_ISL_8601247 | B.1.1.529 | Omicron | USA |
| EPI_ISL_8648443 | B.1.1.529 | Omicron | Israel |
| EPI_ISL_8648509 | B.1.1.529 | Omicron | New Zealand |
| EPI_ISL_8131158 | B.1.1.529 | Omicron | India |
| EPI_ISL_8128493 | B.1.1.529 | Omicron | South Africa |
| EPI_ISL_8133617 | C.37 | Lambda | United Kingdom |
| EPI_ISL_8613336 | C.37 | Lambda | USA |
| EPI_ISL_7476408 | C.37 | Lambda | Colombia |
| EPI_ISL_8189775 | C.37 | Lambda | Peru |
| EPI_ISL_7752590 | C.37 | Lambda | Argentina |
| EPI_ISL_8483100 | B.1.621 | Mu | USA |
| EPI_ISL_2080881 | B.1.621 | Mu | Spain |
| EPI_ISL_431154 | B.1.621 | Mu | France |
| EPI_ISL_8357529 | B.1.621 | Mu | Colombia |
| EPI_ISL_8357500 | B.1.621 | Mu | Colombia |


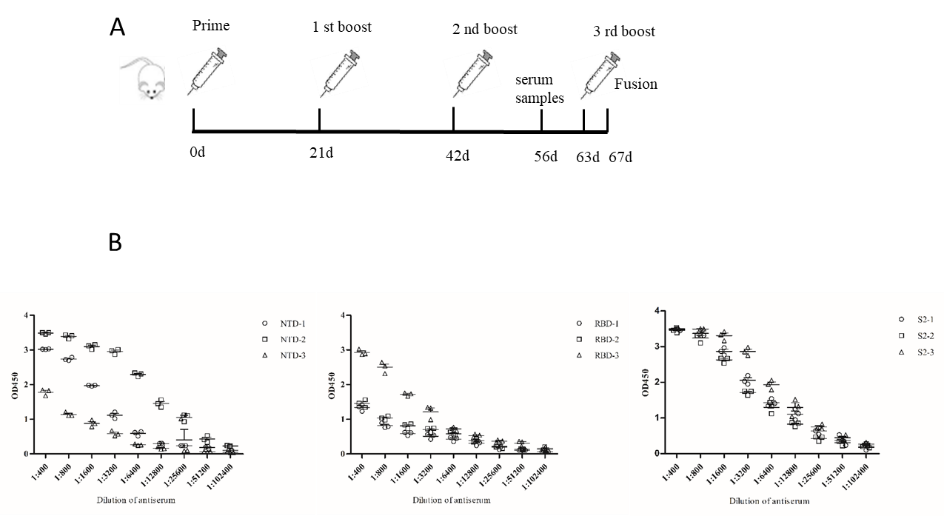


**Figure S1** **Serum antibody titer after immunization in mice.**

(A) Strategy formulation of mice immune antigen proteins. (B) The titers of anti-NTD, RBD and S2 antibodies in serum of mice at 2 weeks after three immunizations were determined by indirect-ELISA.
